# High-Performance Ultraviolet Photodetector Based on Graphene Quantum Dots Decorated ZnO Nanorods/GaN Film Isotype Heterojunctions

**DOI:** 10.1186/s11671-018-2672-5

**Published:** 2018-08-30

**Authors:** Deshuai Liu, Hui-Jun Li, Jinrao Gao, Shuang Zhao, Yuankun Zhu, Ping Wang, Ding Wang, Aiying Chen, Xianying Wang, Junhe Yang

**Affiliations:** 1 School of Materials Science and Engineering, University of Shanghai for Science and Technology, No. 516 JunGong Road, Shanghai, 200093 China; 2 Shanghai Innovation Institute for Materials, Shanghai, 200444 China; 3 Hong Kong Beida Jade Bird Display Ltd, Shanghai, 201306 China

**Keywords:** ZnO nanorod arrays, Graphene quantum dots, Heterojunction, UV photodetector

## Abstract

A novel isotype heterojunction ultraviolet photodetector was fabricated by growing n-ZnO nanorod arrays on n-GaN thin films and then spin-coated with graphene quantum dots (GQDs). Exposed to UV illumination with a wavelength of 365 nm, the time-dependent photoresponse of the hybrid detectors manifests high sensitivity and consistent transients with a rise time of 100 ms and a decay time of 120 ms. Meanwhile, an ultra-high specific detectivity (up to ~ 10^12^ Jones) and high photoresponsivity (up to 34 mA W^−1^) are obtained at 10 V bias. Compared to the bare heterojunction detectors, the excellent performance of the GQDs decorated n-ZnO/n-GaN heterostructure is attributed to the efficient immobilization of GQDs on the ZnO nanorod arrays. GQDs were exploited as a light absorber and act like an electron donor to effectively improve the effective carrier concentration in interfacial junction. Moreover, appropriate energy band alignment in GQDs decorated ZnO/GaN hybrids can also be a potential factor in facilitating the UV-induced photocurrent and response speed.

## Background

UV photodetectors have attracted great attention in the fields of missile launching detection, space and astronomical research, environmental monitoring, UV radiation calibration and monitoring, and optical communication [1]. Semiconductors with wide band gaps are a series of common choices for UV photodetectors, such as GaN [2], CdS [3], ZnO [4, 5], Ga_2_O_3_ [6], ZnS [7], and SiC [8], since they exhibit significant ultraviolet UV absorption. Among them, ZnO nanomaterials have been intensively explored for short-wavelength optoelectronics devices, due to its wide band gap (about 3.37 eV) and high exciton binding energy (about 60 meV) at room temperature [9–12].

Many efforts have been made on constructing ZnO-based UV photodetectors using ZnO single crystals, thin films, or nanostructures [13–15]. Generally, the photodetection and photoresponse performance of ZnO material are key parameters to determine the capability of the UV photodetector, which is related with its surface condition, structural quality, and rate of oxygen adsorption and desorption. Fabrication of one-dimensional ZnO is found to be an efficient solution to improve its photodetection and photoresponse performance. Meanwhile, various nanostructures including heterostructures [16], homojunctions [17], nanocomposites [18, 19], and ZnO of special morphologies [20] have also been sequentially reported which could furtherly shorten the rise and decay time of ZnO-based UV detectors. By comparison, n-ZnO/n-GaN isotype heterojunctions have been proven to be a superior choice owing to their similar crystal structure, lattice parameter, and wide band gaps (3.37 eV for ZnO and 3.39 eV for GaN), which could generate carriers from the interior localized states excited by light or electric field.

Another widely employed material to fabricate ZnO-based heterojunctions is quantum dots (QDs), which contribute to increase the photogenerated charge separation and transportation rate in ZnO nanostructures. The decoration of QDs on ZnO nanostructures can introduce new interfaces and greatly improve charge separation through transferring the electrons from QDs to the conduction band of ZnO, thus leading to the enhancement of photoresponse under ultraviolet light irradiation. Recently, graphene quantum dots (GQDs), a single-layer graphene with a few nanometers in two-dimensional direction, have held promising application prospects as a light-absorbing material in designing broadband photodetectors and photovoltaic devices, attributed to its size-dependent band gap and strong optical absorption [21]. Dhar et al. have prepared a series of GQDs decorated nanorod/polymer Schottky junction UV detector [22–24]. Yang et al. have found that the photocurrent of GQDs coated ZnO nanorod array (ZNRA) illuminated by UV light was enhanced remarkably compared to that of pure nanoarrays. They proposed that this improvement was probably ascribed to the charge transfer at the interface of GQDs and ZNRA [25]. Rahimi et al. have then reported that the incorporation of GQDs on aligned ZnO nanorods yielded faster sensing speed, and the maximum UV-excited photocurrent is ~ 2.75 times higher than that of the bare ZnO thin film [26]. Therefore, it is reasonable to utilize the advantages of GQDs mentioned above to boost the UV sensing properties of ZnO. However, as to our knowledge, there is no reported research that reveals the function of GQDs in n-ZnO nanorod arrays/n-GaN photodetector.

In this paper, n-ZnO/n-GaN isotype heterojunction UV photodetector decorated with GQDs has been fabricated via a facile method. An obvious enhancement of the photocurrent and good reproducibility of the GQDs decorated heterojunction detector has been observed, in contrast to that of the bare n-ZnO/n-GaN detector. The superior photo-to-dark current ratio and response rate of the hybrid UV photodetector can be attributed to the synergistic effect and appropriate energy band structures of n-ZnO, n-GaN, and GQDs, in which GQDs were exploited as the light absorbers and electron donors to greatly boost the electron transport in n-ZnO/n-GaN isotype heterogeneous junction. These efforts broaden the application potential of GQDs in UV photodetectors and pave a new way to explore the various photodetection performances by designing hybrid nanostructures.

## Methods/Experimental

### Preparation of n-ZnO/n-GaN Heterojunction

All the reagents of analytical grade were purchased from Sigma-Aldrich and used as received without further purification. The n-ZnO nanorod arrays/n-GaN film isotype heterojunctions were prepared via a two-step process. Firstly, the n-GaN film was synthesized on Al_2_O_3_ substrate by the metal organic chemical vapor deposition method (MOCVD). Then, the ZnO NRs were directly grown on the n-GaN film by a hydrothermal method which has been reported in previous studies [27]. Firstly, the Al_2_O_3_ substrate plated with n-GaN film was placed in an aqueous solution containing 0.025 M zinc acetate ((CH_3_COO)_2_Zn·2H_2_O) and 0.025 M hexamethylene tetramine (C_6_H_12_N_4_) as the precursors. The precursors were transferred into a Teflon-lined stainless steel autoclave. Next, the autoclave was sealed and put into the oven. The hydrothermal treatments were carried out at 95 °C for 12 h. Finally, the autoclave was allowed to cool down naturally. The samples were taken out, washed using deionized water for several times, and dried in air.

### Synthesis of GQDs

The graphene quantum dots were prepared via a hydrothermal method utilizing pyrolyzed citric acid (CA) as the precursor in an alkaline environment according to some previously reported literature [28–30]. Typically, 0.21 g (1 mmol) CA and 0.12 g (3 mmol) sodium hydroxide (NaOH) were dissolved into 5 mL water and stirred to form a clear solution. Then, the solution was transferred into a 20-mL Teflon-lined stainless autoclave. The sealed autoclave was heated to 160 °C in an electric oven and kept for additional 4 h. The synthesized GQDs were collected by adding ethanol into the solution and centrifuged at 10000 rpm for 5 min and then ultrasonic cleaned with ethanol for three times. The solid can be easily re-dispersed into water.

### Fabrication of UV Photodetector

**Scheme 1:**
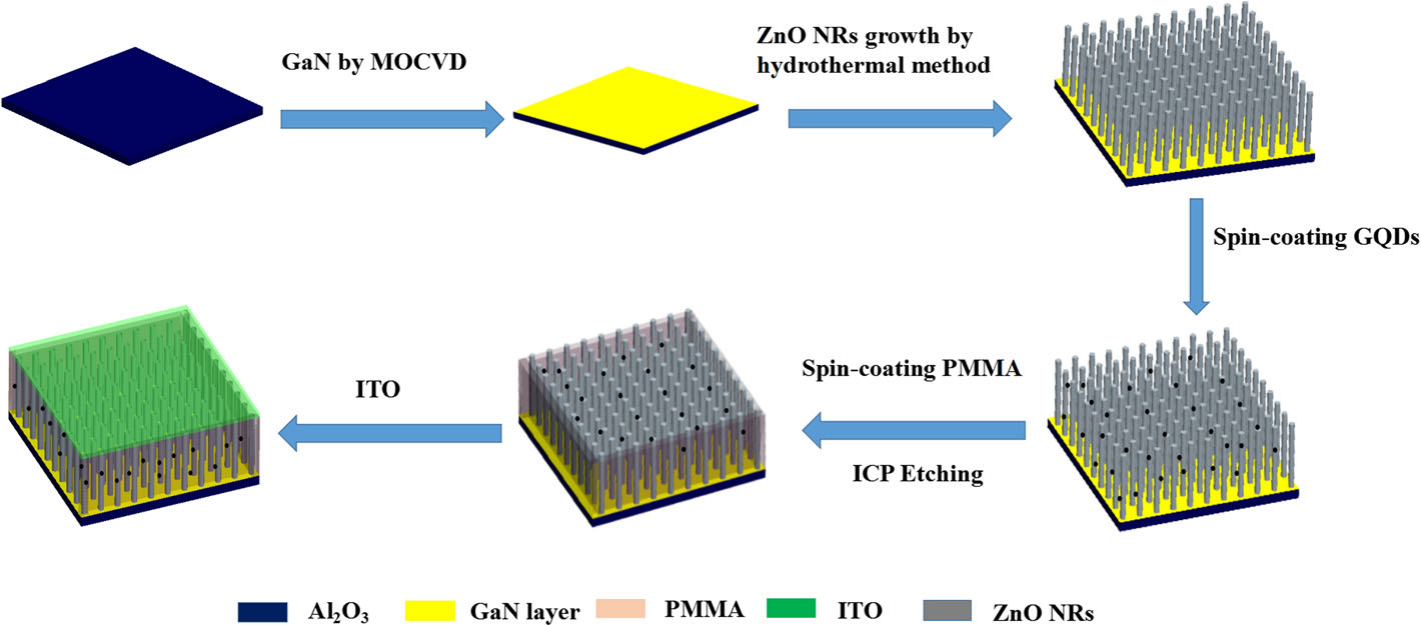
Schematic diagram of the fabrication process of the isotype heterojunction UV photodetector

The Al_2_O_3_ substrate plated with n-ZnO/n-GaN heterojunction was firstly cleaned with deionized water and ethanol and dried at 60 °C in air. Then, the GQDs were spin-coated on the heterojunctions. After that, the devices were spin-coated with polymethylmethacrylate (PMMA), followed by inductively coupled plasma (ICP) etching. The devices were covered by the indium tin oxide (ITO) immediately, and an Ag electrode was applied on GaN for Ohmic contacts. The final effective area of the isotype heterojunction is ~ 5 × 5 mm^2^. A schematic diagram of the fabrication process of the n-ZnO nanorod arrays/n-GaN film isotype heterojunction is shown in Scheme 1.

### Characterization

The surface morphology of the ZnO nanorod arrays was characterized using the field-emission scanning electron microscope (FE-SEM, FEI, Quanta FEG). The morphology and size distribution of the GQDs was characterized by high-resolution transmission electron microscope (HRTEM, FEI, Tencai G20). The UV-vis spectra were recorded on a Lambda 25 UV-vis spectrophotometer (PerkinElmer, USA). The photoluminescence spectroscopy (PL) was recorded using a Shimadzu RF-5301 Fluorescence spectrophotometer. X-ray photoelectron spectroscopy (XPS) was performed using a ThermoFisher-250XI X-ray electron spectrometer with focused monochromatized Al Kα radiation. The crystal structures were measured using X-ray diffractometer (XRD, Brukes, D8 Advance). Raman spectra were examined using Raman station 400F machine (PerkinElmer). The photocurrent response was measured by a semiconductor characterization system (Keithley 4200), and a 300 mW/cm^2^ Xenon lamp (365 nm) was employed as the UV light irradiation source.

## Results and Discussions

**Fig. 1:**
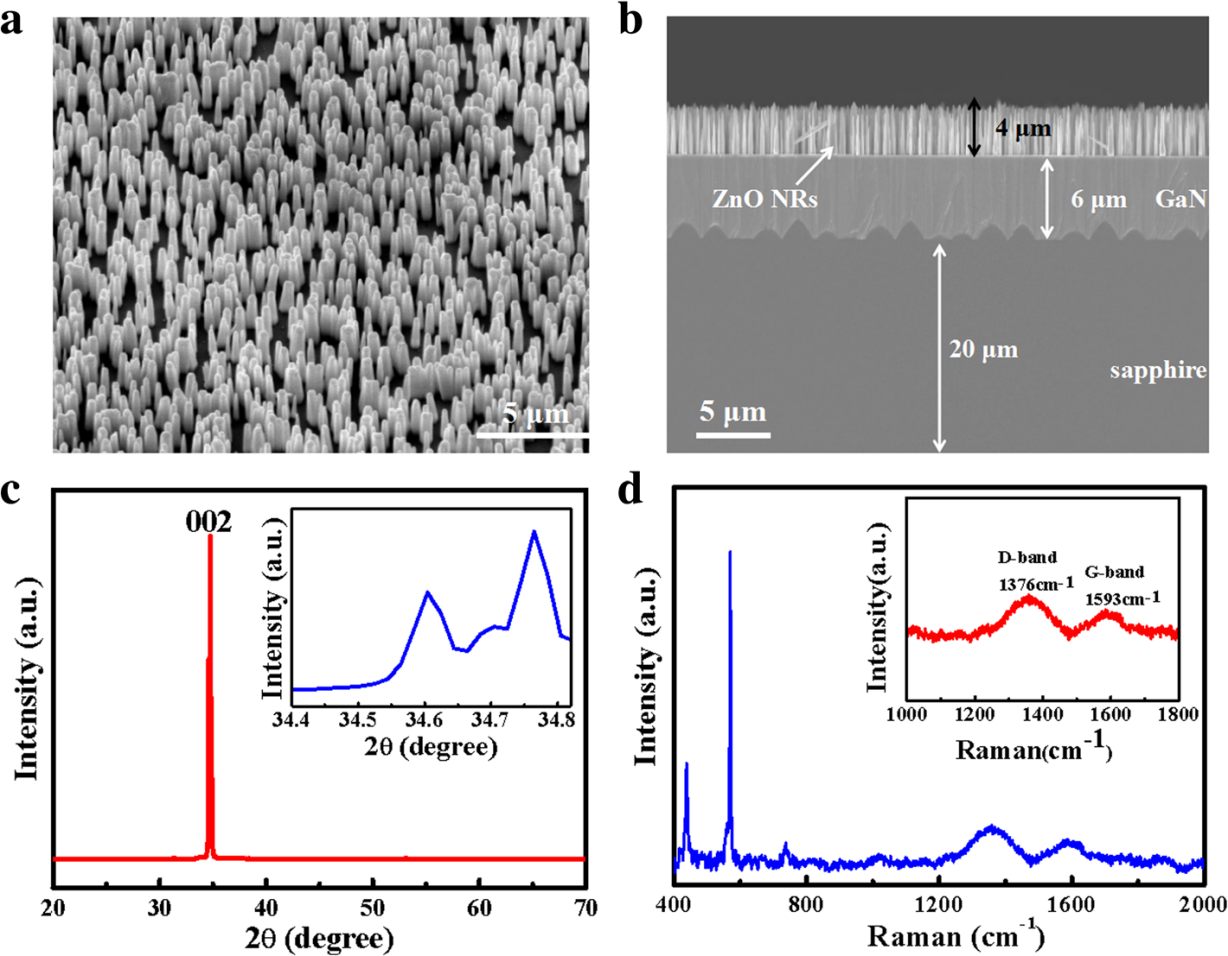
**a** The FE-SEM image of ZnO nanorod arrays grown over GaN film on Al_2_O_3_ substrate (45° tilted). **b** The cross-sectional FE-SEM image of the device. **c** The X-ray diffraction pattern of ZnO/GaN sample (inset: high resolution rocking curve of the (002) reflection resolving ZnO and GaN peaks). **d** Raman spectra of n-ZnO/n-GaN heterojunctions decorated with GQDs

Figure 1a presented the SEM image of the as-grown ZnO nanorod arrays. Uniform ZnO nanorod arrays on entire Al_2_O_3_ substrate plated with GaN film have been obtained under hydrothermal conditions. Figure 1b shows the cross-sectional SEM image of the device. The thickness of the substrate, GaN film, and ZnO NRs is measured as 20, 6, and 4 μm, respectively. Figure 1c depicts the X-ray diffraction pattern of n-ZnO/n-GaN heterojunctions. GaN and ZnO with wurtzite crystal structure have similar lattice parameters, thus leading to merge of the (002) diffraction peaks of the two semiconductors. Through analysis of the high-resolution X-ray rocking curve, the (002) peaks of both GaN and ZnO could be observed clearly, shown in the inset of Fig. 1c. The strongest (002) diffraction peak indicated that the microrods mainly grow along the [001] direction. In Fig. 1d, the D band at ≈ 1360 cm^−1^ and G band at ≈ 1600 cm^−1^ could also be observed, which are attributed to the sp^2^ graphitized structure and local defects/disorders of carbonaceous materials, respectively. The high ratio of D/G peak intensity demonstrated that large amounts of defects and disorders existed in the edge or surface of the GQDs structure [31].

**Fig. 2:**
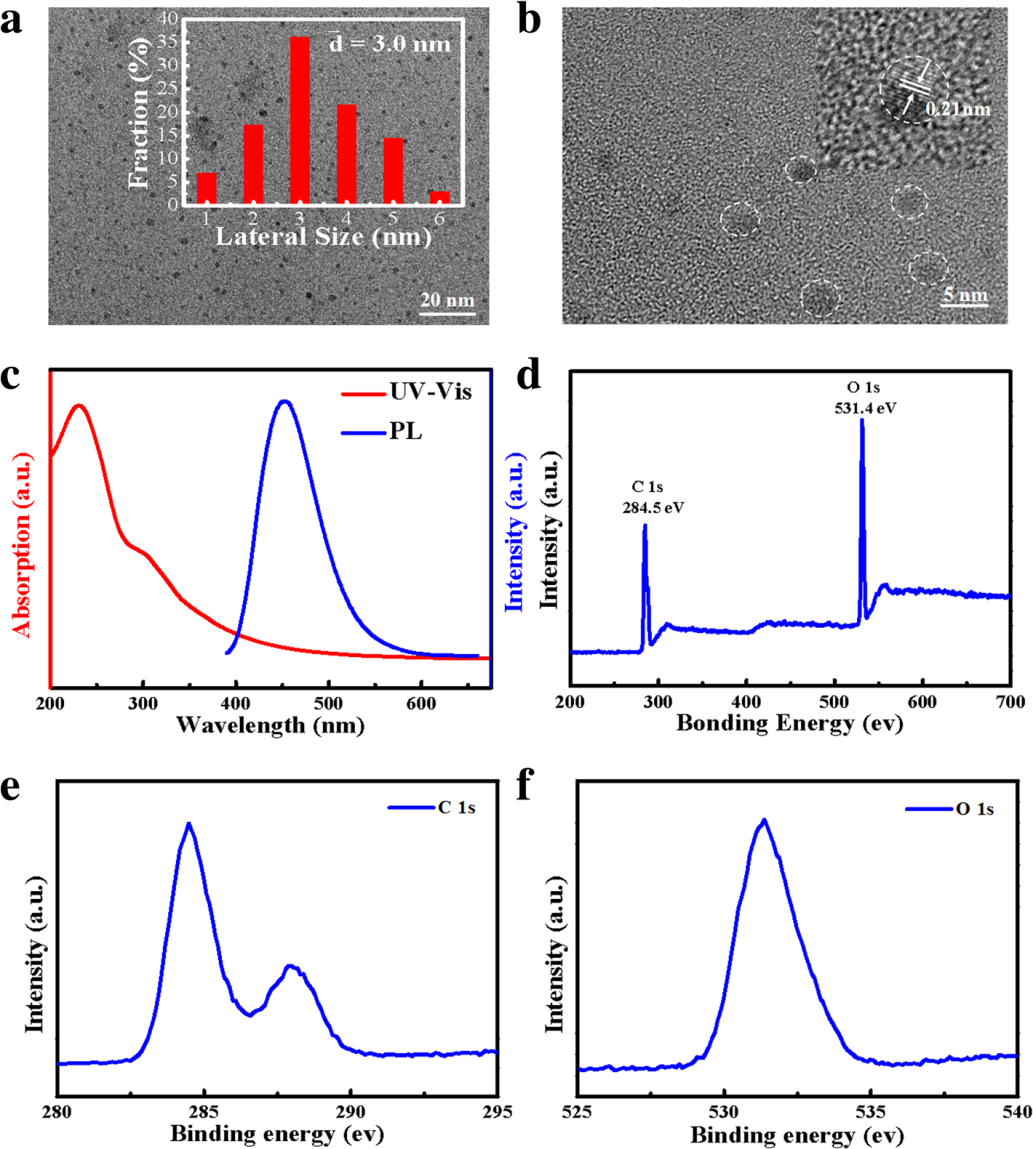
**a** TEM image (inset: size distribution of GQDs). **b** HRTEM image of GQDs. **c** UV-vis spectra and PL spectra of the GQDs (the excitation wavelength is 365 nm). **d** XPS survey spectra. **e** C 1s high-resolution XPS spectra. **f** O 1s high-resolution XPS spectra

Figure 2a, b shows the TEM and HRTEM images of the obtained GQDs. It can be found that the GQDs have a relatively uniform particle size distribution with a lattice fringe of 0.21 nm, and the average lateral size was statistically calculated to be 3.0 ± 0.6 nm (seen from the inset in Fig. 2a). Figure 2c shows the UV-Vis spectrum of the GQDs. As can be seen, there is a strong peak around 240 nm, corresponding to the π–π* transition of aromatic sp^2^ clusters, and a weaker shoulder in the range of 300~320 nm, corresponding to the n–π* transition of C=O bonds [32, 33]. The PL spectra of the GQDs exhibit a peak centered at 442 nm, mainly originated from π→π* transition. In the XPS survey spectrum, two peaks centered at ~ 284.5 eV and 531.4 eV were shown in Fig. 2d, which corresponds to C 1s and O 1s, respectively. The high-resolution C 1s spectrum demonstrates two peaks at 284.8 and 288.7 eV (Fig. 2e). The binding energy peak at 284.8 eV is ascribed to C=C bonds, and the binding energy peak at 288.7 eV is attributed to O=C–O bonds. The high-resolution O 1s spectrum of the sample (Fig. 2f) shows a peak at 531.8 eV, attributed to the C=O group [34]. The analysis indicates that the basic structure of the GQD sample is aromatic unit, similar to some previous literatures [35].

**Fig. 3:**
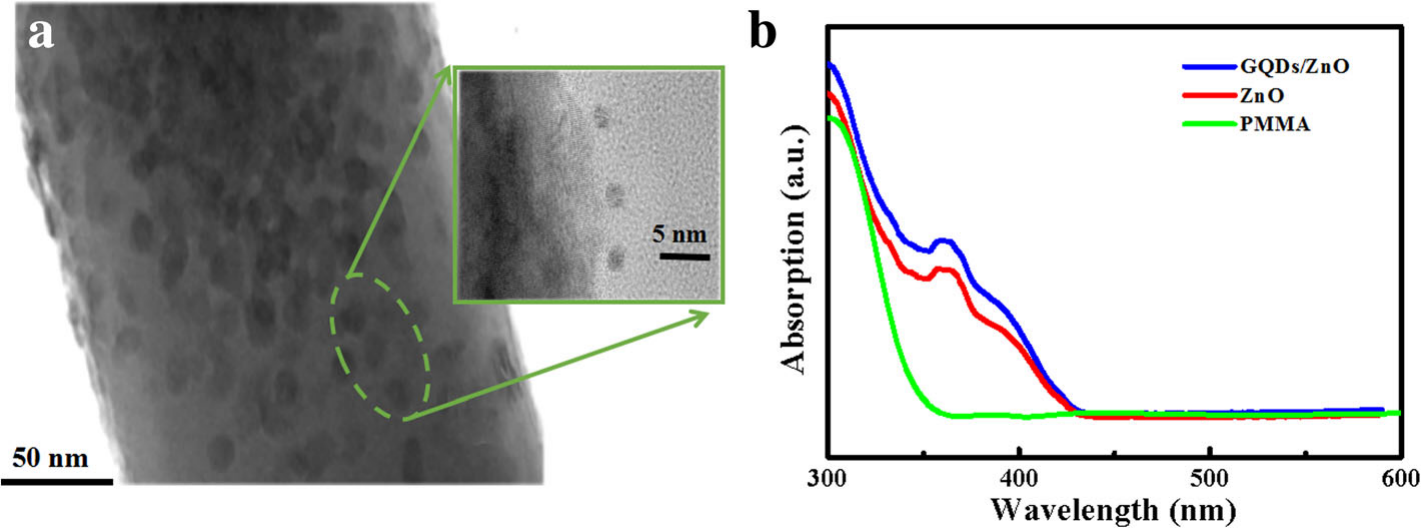
**a** TEM image of a representative GQDs/ZnO nanorod (inset: HRTEM image of the green circle in (**a**)). **b** UV-DRS absorption spectra of the GQDs/ZnO nanorods, bare ZnO nanorods, and PMMA

To furtherly examine the GQDs decorated heterojunction nanoarrays, TEM image of a representative GQDs/ZnO nanorod was shown in Fig. 3a, demonstrating a uniform decoration of GQDs on the ZnO nanorods. The inset in Fig. 3a corresponds to the HRTEM image circled by a green square. The UV-DRS spectra of the ZnO nanorods decorated with/without GQDs have also been compared, shown in Fig. 3b. The devices show a strong absorption in the ultraviolet region. Furthermore, the light absorption intensity of the ZnO nanorod array decorated with GQDs is enhanced by a factor of approximately 20%, compared to that of the bare ZnO nanorods. The higher UV absorption of the GQDs treated ZnO nanorods makes the device more suitable when applied in UV photodetectors. Meanwhile, the pure PMMA mainly absorbs light in the range of 300~350 nm, shown in Fig. 3b. In our study, the UV light irradiation source is 365 nm; thus, the effect of PMMA on the photoresponse performance of the whole device is negligible.

**Fig. 4:**
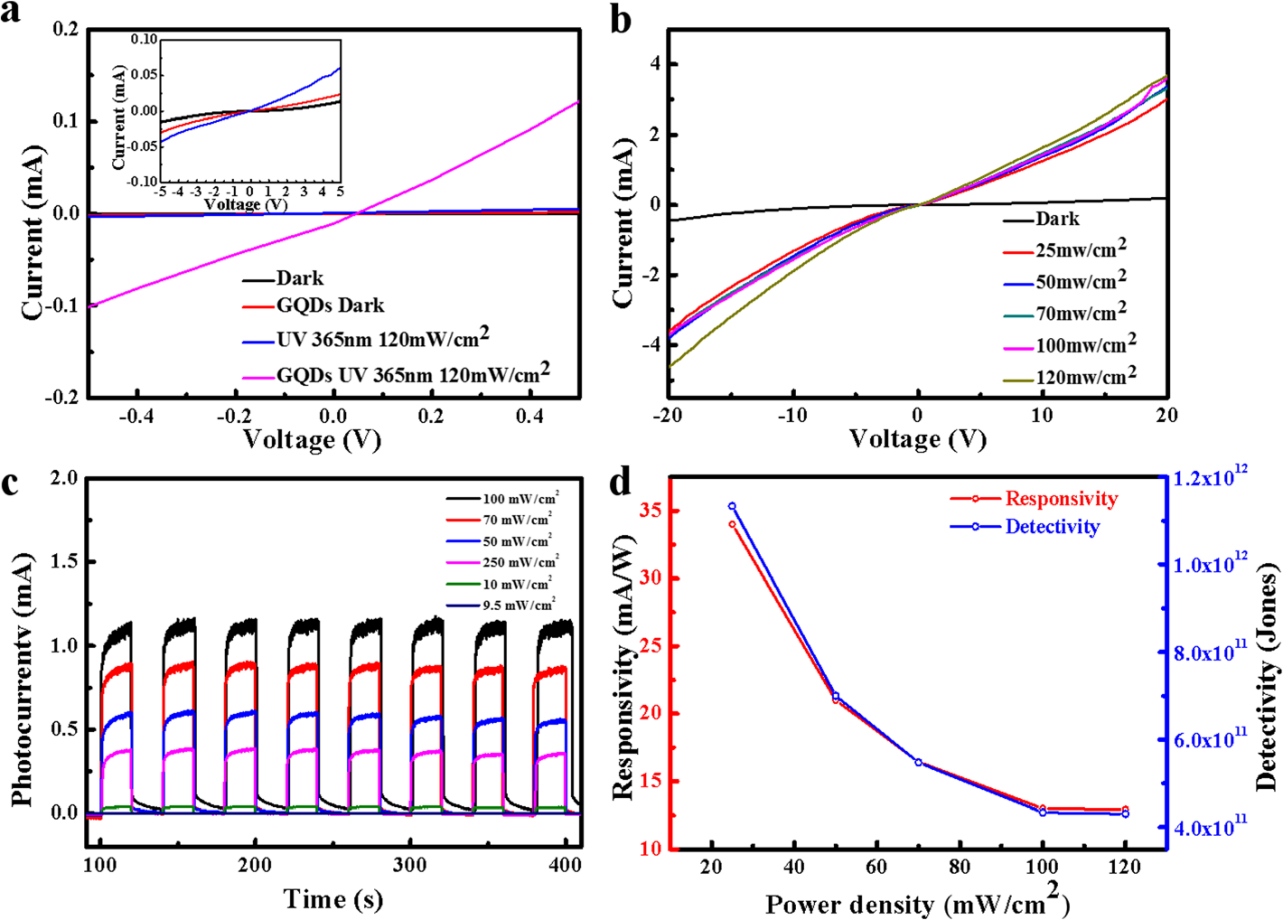
**a** The I–V characteristic curves of the UV photodetectors under dark and UV light irradiation decorated with/without GQDs (inset: the magnified I–V characteristic curves of the UV photodetectors). **b** The I–V characteristic curves illuminated with UV light of different incident power densities (mW/cm^2^). **c** The photoresponse at different incident light power densities (mW/cm^2^). **d** The responsivity (red) and detectivity (blue) as a function of the incident light power density, respectively

Figure 4a, b plots the I–V characteristics curves of the ZnO NRs/GaN UV photodetectors decorated with and without GQDs under dark (power density = 0 mW/cm^2^) and UV illumination (*λ* = 365 nm, power density = 120 mW/cm^2^), respectively. In dark, the I–V characteristic curve exhibits a typical rectifying characteristic with a very low leakage current, and the current increases linearly with the applied voltage shown in the inset of Fig. 4a, signifying the Ohmic contact between the heterojunction and the electrodes, while the dark current increases slightly by coating the heterojunction with GQDs. When irradiated under UV light, the photocurrent of the photodetector decorated without GQDs nearly kept the same. However, the photocurrent of the device coated with GQDs increases dramatically and reaches a large value of 0.4 mA at the applied bias of 1.5 V, which is more than 40 times higher than its corresponding dark current.

In addition, we examined the photoresponse of ZnO/GaN UV photodetectors under 365 nm UV light illumination at 10 V bias. Figure 4c displays the time-dependence of the photocurrent with respect to incident power densities of 9.5, 10, 25, 50, 70, and 100 mW/cm^2^. It can be found that when the incident power density is 9.5 mW/cm^2^, the light current of the device showed no response. Meanwhile, the minimum accuracy of the UV lamp is 0.5 mW/cm^2^. Therefore, we can infer that the minimum light intensity detected by the device is among 9.5~10 mW/cm^2^. The photocurrent increased upon increasing the light power density and changed instantly in response to on/off switching cycles of the light source. The reversible and reproducible switching revealed good stability of the devices. Moreover, the performance of the photodetector can be quantified by the responsivity (*R*_λ_), defined as [25],

$$ {R}_{\lambda }=\frac{I_{\mathrm{ph}}}{P_{\mathrm{opt}}} $$

where *I*_ph_ is the difference between the currents measured under illumination with light and in dark, *P*_opt_ is the incident power of the device, and *λ* is the excitation light wavelength. The calculated responsivities of the device under incident power densities of 25, 50, 70, 100, and 120 mW/cm^2^ were 34, 21, 16.4, 13, and 12.9 mA/W, respectively.

Figure 4d shows the responsivity of the photodetector as a function of incident power density. The device is very sensitive to UV light illumination. With the increase of illumination light power, the detectivity and responsivity decrease obviously, which might be owing to the absorption saturation of ZnO or the screening of the built-in electric field by the photoexcited electrons in the conduction band of ZnO [36]. Assuming that short noise from the dark current is the major noise source, the specific detectivity (D*) can be expressed as [37]:

$$ {D}^{\ast }=\frac{R_{\lambda }}{{\left(2e\cdot {I}_{\mathrm{dark}}/S\right)}^{1/2}} $$

where *e* is the charge of an electron and *I*_dark_ is the dark current. Accordingly, the maximum detectivity up to 10^12^ Jones has been achieved, which is higher than that of the photodetectors based on most ZnO photodetectors [38, 39]. The employment of GQDs as the light absorbers and electron donors could attribute to enhancement of carrier concentration in heterogeneous junction, thus greatly improving the responsivity and detectivity of the UV photodetectors.

**Fig. 5:**
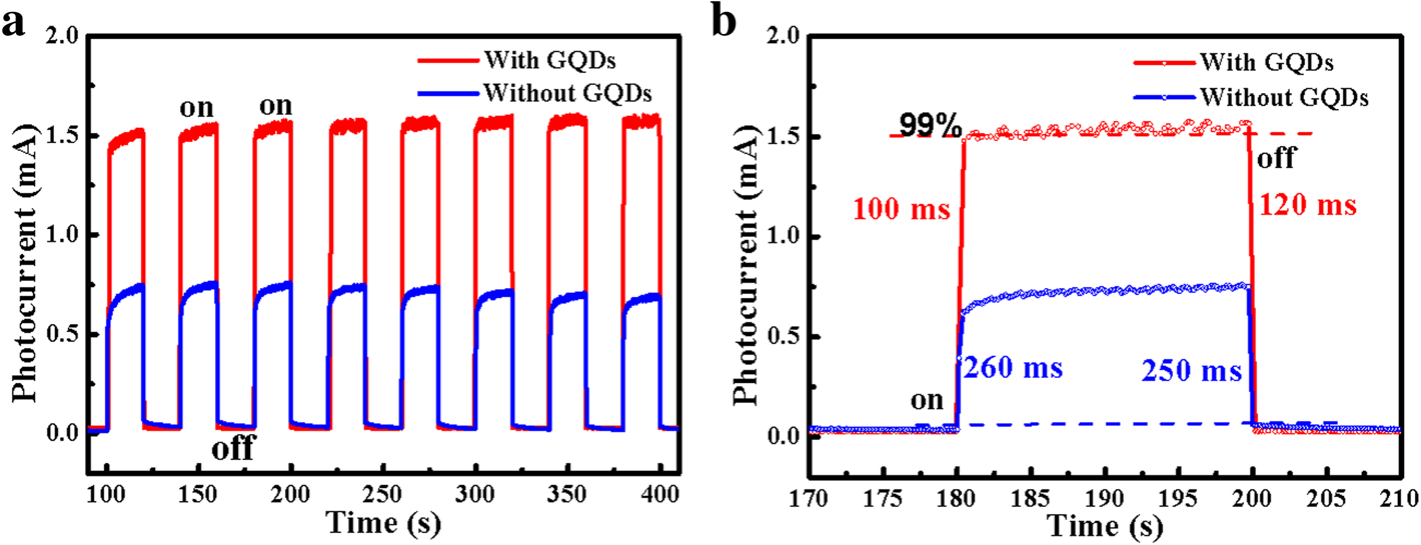
**a** The reproducible on/off switching of the device decorated with/without GQDs upon 365 nm light illumination with a 20-s cycle under 10 V bias, respectively. **b** The enlarged portions of the light-off to light-on and light-on to light-off transitions with/without GQDs decoration, respectively

To examine the response rate and stability of the n-ZnO/n-GaN UV photodetectors decorated with GQDs, the time-resolved photocurrent at 10 V bias with multiple on/off cycles has been measured. As shown in Fig. 5a, the photocurrent of the device exhibits two distinct states, a low-current state in dark and a high-current state under 365 nm UV light illumination. The current increases sharply from one state to another, indicative of a very fast response rate of the two samples. As shown in Fig. 5b, the time-resolved photocurrent revealed that the response rate of the ZnO UV photodetectors decorated with GQDs is faster than that of the bare one. In view of the process, the current would rapidly ramp to the saturated value upon UV illumination. The rise times corresponding to the heterojunction photodetectors decorated with and without GQDs were ~ 100 ms and ~ 260 ms, respectively. When the light is off, the photocurrent promptly falls to the dark current value after ~ 120 ms and ~ 250 ms which correspond to the ZnO NRs/GaN UV photodetectors decorated with and without GQDs, respectively. The response rate in our studies is comparable or even faster than many reported results, shown in Table 1.

**Table 1:** Comparison of the characteristic parameters of the isotype heterojunction UV photodetector

Material	Substrate	Bias (V)	Wavelength (nm)	Rise time (s)	Decay time (s)	References
ZnO film/GQDs	Glass	0	UV	2.6	6.31	[26]
ZnO nanorods/CdS	GaN	0	254	< 0.35	< 0.35	[44]
ZnO nanorods	GaN	1	360	0.28	0.32	[45]
ZnO nanorods/GQDs	FTO	2	365	2.14	0.91	[46]
ZnO nanorods/ZnO film	GaN	− 4	362	< 1	< 1	[47]
ZnO nanorods arrays	GaN	10	365	0.26	0.25	This work
ZnO nanorods arrays/GQDs	GaN	10	365	0.1	0.12	This work

**Scheme 2:**
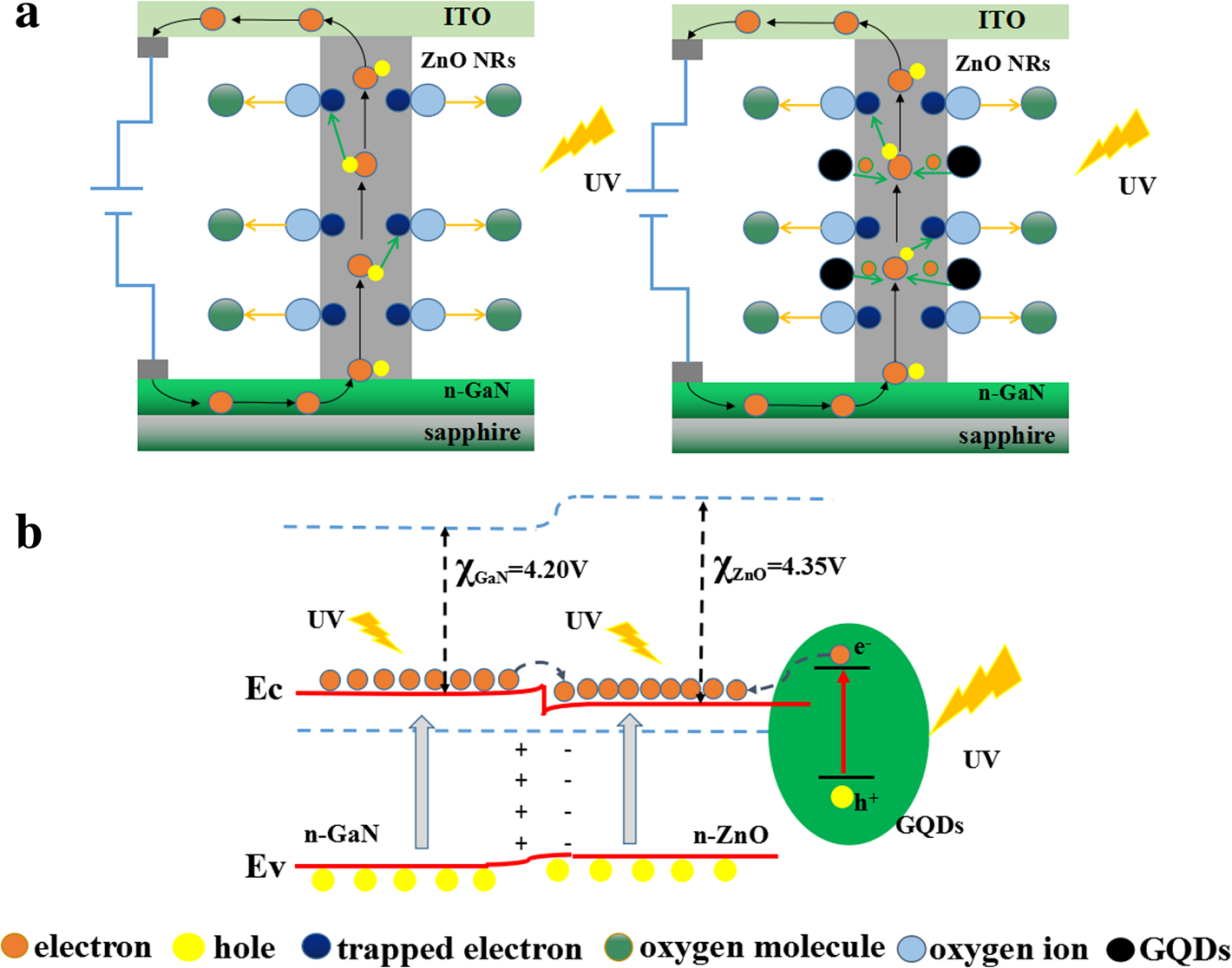
**a** The schematic diagrams of the ZnO NRs/GaN UV photodetector decorated without and with GQDs. **b** Energy band diagram of the GQD-ZnO NRs composite and its carrier transport mechanism in the interfacial region irradiated by UV light

The schematic diagrams of the photoresponse mechanism for the UV photodetector are illustrated in Scheme 2. Surface oxygen on ZnO nanorods is a crucial factor in influencing the observed photoresponse. As is shown in Scheme 2a, the electron capture process is mainly mediated by the oxygen adsorption and desorption process at the ZnO NRs surface under ambient circumstances. The absorbed oxygen molecules firstly capture free electrons from the ZnO NRs, leading to the formation of depletion layer near the surface and charged oxygen ions (O_2_^−^). The depletion layer decreases the conductivity of ZnO NRs. When the ZnO NRs were illuminated by 365 nm UV light with the energy level above or close to the band gap of ZnO, the electron–hole pairs generate. After that, most of the photogenerated holes are rapidly trapped by oxygen ions (O_2_^−^), resulting in the discharge of oxygen ions and desorbing of the oxygen from ZnO surface. The hole-capturing process attributes to the increase of free-carrier concentration, producing an apparent enhancement in conductivity. When the UV irradiation is switched off, the holes recombine with electrons, and oxygen re-adsorbed onto ZnO nanorods again. The photoresponse mechanism for the n-ZnO/n-GaN UV photodetector decorated with GQDs is similar while more electrons would generate if the ZnO NRs were coated with GQDs.

Scheme 2b displays the band diagram of the GQDs-ZnO/GaN composite and its carrier separation/transport mechanism in the interfacial region under UV irradiation. The band gap of ZnO is around − 3.27 eV, and its conduction band is located at − 4.35 eV below the vacuum level [40]. The band gap of n-GaN is around − 3.39 eV, and its conduction band is located at − 4.20 eV below the vacuum level [41]. When the two semiconductors are contacted, an energy barrier of 0.15 eV appears between the two conduction bands (Δ*E*_*c*_). The HOMO and LUMO position of the GQDs were obtained from the literature in which the GQDs were prepared via the same method [42]. The band gap of GQDs is around 1.5 eV with its LUMO band of − 3.5~3.7 eV and HOMO band of − 5.1~5.4 eV versus vacuum level [43]. The CB band level of GaN and GQDs is higher than that of ZnO, while the VB band level of ZnO is higher than that of GaN and GQDs. Therefore, when ZnO is decorated with GQDs irradiated under UV light, the bands of GaN and GQDs will bend downward and the bands of ZnO will bend upward near the interface. Then, the photogenerated electrons on the conduction band of GaN and GQDs can be efficiently transferred to the conduction band of ZnO. Compared to the majority carrier, the movement of the holes in the valence band of n-GaN and n-ZnO can be neglected. As a result, there is a significant increase of unpaired electrons upon UV illumination which could contribute to the enhancement of carrier injection and transportation and thus dramatically increasing the photocurrent. During this process, the rapid separation of photogenerated electron–hole pairs and efficient carrier migration is responsible to the fast responding rate.

## Conclusions

The photocurrent and sensing rate of GQDs decorated n-ZnO/n-GaN heterojunctions illuminated under UV light is enhanced remarkably compared to that of pure n-ZnO/n-GaN detectors. The maximum photocurrent of the hybrid device reaches 0.4 mA at the applied bias of 1.5 V, which is more than 40 times higher than its corresponding dark current. The device showed selective UV response with pulse duration within milliseconds. The superior performance of the ZnO/GaN heterostructures is attributed to the efficient immobilization of GQDs on ZnO NRs which function as the light absorbers and electron donors, and also appropriate energy band alignment in GQDs decorated ZnO/GaN hybrids. The designing device holds the prospects for utilizing the synergistic effect of multi-composites, paving the way for developing GQD-sensitized efficient optoelectronic n-type devices.

## Abbreviations

FE-SEM: Field-emission scanning electron microscope
GQDs: Graphene quantum dots
HR-TEM: High-resolution transmission electron microscopy
ICP: Inductively coupled plasma
ITO: Indium tin oxide
MOCVD: Metal-organic chemical vapor deposition
PMMA: Polymethylmethacrylate
XPS: X-ray photoelectron spectroscopy
XRD: X-ray diffractometer
ZNRA: ZnO nanorod array
